# The testis-specific serine proteases PRSS44, PRSS46, and PRSS54 are dispensable for male mouse fertility^†^

**DOI:** 10.1093/biolre/ioz158

**Published:** 2019-08-12

**Authors:** Richard J Holcomb, Seiya Oura, Kaori Nozawa, Katarzyna Kent, Zhifeng Yu, Matthew J Robertson, Cristian Coarfa, Martin M Matzuk, Masahito Ikawa, Thomas X Garcia

**Affiliations:** 1 Department of Pathology and Immunology, Baylor College of Medicine, Houston, TX, USA; 2 Department of Biology and Biotechnology, University of Houston-Clear Lake, Houston, TX, USA; 3 Center for Drug Discovery, Baylor College of Medicine, Houston, TX, USA; 4 Department of Experimental Genome Research, Research Institute for Microbial Diseases, Osaka University, Suita, Osaka, Japan; 5 Dan L. Duncan Comprehensive Cancer Center, Baylor College of Medicine, Houston, TX, USA; 6 Advanced Technology Cores, Baylor College of Medicine, Houston, TX, USA; 7 Department of Molecular and Cellular Biology, Baylor College of Medicine, Houston, TX, USA; 8 Department of Molecular and Human Genetics, Baylor College of Medicine, Houston, TX, USA; 9 Department of Pharmacology and Chemical Biology, Baylor College of Medicine, Houston, TX, USA; 10 Graduate School of Pharmaceutical Sciences, Osaka University, Suita, Osaka, Japan; 11 The Institute of Medical Science, The University of Tokyo, Minato-ku, Tokyo, Japan

**Keywords:** contraception, CRISPR/Cas9, drug target, male reproductive tract, paralog, sperm maturation, spermatid, spermatozoa

## Abstract

High-throughput transcriptomics and proteomics approaches have recently identified a large number of germ cell–specific genes with many that remain to be studied through functional genetics approaches. Serine proteases (PRSS) constitute nearly one-third of all proteases, and, in our bioinformatics screens, we identified many that are testis specific. In this study, we chose to focus on *Prss44*, *Prss46*, and *Prss54*, which we confirmed as testis specific in mouse and human. Based on the analysis of developmental expression in the mouse, expression of all four genes is restricted to the late stage of spermatogenesis concomitant with a potential functional role in spermiogenesis, spermiation, or sperm function. To best understand the male reproductive requirement and functional roles of these serine proteases, each gene was individually ablated by CRISPR/Cas9-mediated ES cell or zygote approach. Homozygous deletion mutants for each gene were obtained and analyzed for phenotypic changes. Analyses of testis weights, testis and epididymis histology, sperm morphology, and fertility revealed no significant differences in *Prss44*, *Prss46*, and *Prss54* knockout mice in comparison to controls. Our results thereby demonstrate that these genes are not required for normal fertility in mice, although do not preclude the possibility that these genes may function in a redundant manner. Elucidating the individual functional requirement or lack thereof of these novel genes is necessary to build a better understanding of the factors underlying spermatogenesis and sperm maturation, which has implications in understanding the etiology of male infertility and the development of male contraceptives.

## Teaser

**Summary Sentence** The testis-specific serine proteases *Prss44, Prss46*, and *Prss54* are dispensable for male fertility based on phenotype analyses of knockout mice produced using the CRISPR/Cas9 system.

## Introduction

Spermatogenesis is a complex process through which haploid spermatozoa are produced from immature diploid germ cells in the seminiferous epithelium of the testis in mammals [1]. Various reproductive tract specific or ubiquitously expressed proteases have been shown to serve vital roles during spermatogenesis and sperm development. For example, cysteine proteases, aspartic acid proteases, metalloproteases, and ubiquitin proteasome-related genes that include ubiquitin-ligases and deubiquitinases are specifically expressed in spermatogenic cells and appear to be involved in multiple steps of spermatogenesis [2]. The murine genes *Ace*, *Adam1a*, *Adam2*, *Adam7*, *Adam24*, *Adamts2*, *Cstl*, *Usp2*, *Usp14*, *Uchl1*, and *Cyld* were shown to be crucial for proper sperm development by a number of forward and reverse genetic approaches, specifically during progression into meiotic phase, acrosome biogenesis, sperm motility, and apoptosis of germ cells [3–12]. The activity of various reproductive tract-specific proteases may posttranscriptionally regulate the quantity of germ cell components at different stages of sperm development [13]. Their significance also pertains to Sertoli–Sertoli and Sertoli–germ cell interactions in germ cell movement through the seminiferous epithelium during different stages of spermatogenesis [2,14].

It is estimated that more than 2300 expressed genes are germ cell specific within the male reproductive tract [15]. Within this large quantity of expressed genes, many are known to be required for male fertility [16,17]. Serine proteases constitute nearly one-third of all proteases [18]. A subclass of proteases, trypsin-like serine proteases, can be distinguished from others due to the presence of a nucleophilic catalytic serine residue at the active site [19]. The role of this active site is to selectively hydrolyze peptide bonds C-terminal to basic amino acid residues for the purposes of posttranslational modification of polypeptides [20]. It has been shown that several testis-specific, trypsin-like serine proteases play vital roles in the male fertilization process such as spermatogenesis and sperm maturation. Mice lacking *Prss37* (an inactive serine protease) and *Prss55* display sterile phenotypes primarily due to severe sperm motility defects that prevent sperm migration through the oviducts [21,22]. In addition, the authors found a limited capability of sperm–egg binding. Although the mechanism is not fully understood, knockout models of these two genes exhibited a lack of ADAM3, a cell surface protein necessary for cell adhesion activity, which indicated that PRSS37 and PRSS55 are essential for the development of ADAM3 [21,22]. PRSS37 is mainly expressed at steps 9–14 of spermiogenesis, indirectly affecting the posttranslational modifications of ADAM3 during epididymal transit [21]. It likely affects the posttesticular proteolytic cleavage of Adam3 precursor through an intermediate(s), which might be a novel protein yet to be determined [21]. PRSS55 is exclusively expressed during a short time frame during late spermiogenesis and does not exist in mature mouse sperm [22]. Found essential for male fertility in mice [22], the role of PRSS55 was suggested to include the maturation of ADAM3 in sperm and the expression of multiple genes [22]. This role is disputed, however, since a different mouse model lacking *Prss55* showed normal fertility with no detectable defects in testicular histology or sperm counts [23]. A mutant mouse model of another serine protease, PRSS21, exhibited a subfertile phenotype due to decreased motility, morphological malformation, and susceptibility to decapitation as they transit through the epididymis [24]. Due to the proteolytic capability and cell membrane localization of PRSS21, it is hypothesized that this protease is involved in proteolytic cleavage of substrates necessary for the sperm maturation process [24]. While the functional requirement of these proteases has been elucidated, there is an abundance of other serine proteases that still require further investigation. HUGO Gene Nomenclature Committee currently classifies 63 genes as members of the serine protease gene group. Of these, several have already been knocked out in the mouse. It was determined that *Prss16, Prss27*, *Prss35*, and *Tmprss4* have no abnormal phenotype [25–27] and *Prss1*, *Prss8*, *Prss12*, *Prss56*, *Tmprss2*, *Tmprss3, Tmprss6*, *Tmprss11a*, and *Tmprss13* have non-reproductive phenotypes [27–35]. Although a functional requirement for *Prss41* has not been determined through a mouse model, it has been shown to be testis-specific, predominantly expressed in the plasma membrane of spermatogonia and Sertoli cells in the basal compartment, and exhibits an intracellular localization in spermatocytes and spermatids in the adluminal compartment of the seminiferous tubules [36]. In our studies, described below, we identified *Prss44*, *Prss46*, and *Prss54* as testis-specific genes in mouse and human. Based on the analysis of developmental expression in the mouse, expression of all three genes is restricted to the late stage of spermatogenesis concomitant with spermiogenesis and spermiation. Utilizing CRISPR/Cas9, we generated knockout mouse models and determined that each gene is individually dispensable for male fertility in mice.

## Materials and methods

### Ethics statement

Mice were housed in accordance with NIH guidelines and all animal experiments were approved by the Institutional Animal Care and Use Committee (IACUC) at Baylor College of Medicine and Osaka University.

### High-throughput screening to identify testis-specific genes for functional analysis

Sequences for different tissues were downloaded from the Sequence Read Archive (SRA), trimmed using TrimGalore, and aligned against the human genome (GRCh38) or mouse genome (GRCm38) using HISAT2. Gene expression in each tissue was quantified using feature Counts, and tissues were batch corrected by removing unwanted variation using RUVR. Differential gene expression was determined for each nonreproductive tissue against each reproductive tissue using EdgeR. The data comprised 5 human testis datasets [37], 18 purified human germ cell datasets [38], 6 human epididymis segment datasets [39], 9 mouse testis datasets [40], and 6 purified mouse germ cell datasets [41]. An additional 118 datasets contributed to the 26 nonreproductive human tissues [37] and 62 datasets contributed to the 14 nonreproductive mouse tissues [40]. The SRA value for each dataset is listed in Supplementary Table S1.

### Reverse transcription PCR

RNA was isolated from the tissues using TRIzol/chloroform extraction method followed by RNeasy Mini kit from Qiagen with on-column DNase (Qiagen) treatment using the manufacturer’s protocol. RNA was reverse transcribed to cDNA using SuperScript III Reverse Transcriptase from ThermoFisher according to the manufacturer’s protocol. cDNA was then polymerase chain reaction (PCR) amplified using gene-specific primers designed using NCBI primer design tool*.* Primer sequences are listed in Supplementary Table S2. Mouse samples (testis, caput, corpus, cauda, ovary, uterus, and 17 nonreproductive tissue types [adipose, bladder, brain, colon, eye, heart, kidney, liver, lung, prostate, skeletal muscle, skin, small intestine, spleen, stomach]) were obtained from dissection of B6/129 mice; the remaining 2 nonreproductive tissues (smooth muscle and thyroid) were obtained as purified RNAs from Takara Bio (Kusatsu, Japan). Human samples (testis, caput, corpus, and cauda tissues [kindly provided by Robert Sullivan at Université Laval], 8 nonreproductive tissue types (kidney, liver, lung, skin, spleen, and stomach) and one reproductive tissue [ovary]) were obtained from the Baylor College of Medicine Tissue Acquisition and Pathology Core, and the remaining 13 nonreproductive tissue types (adipose, adrenal gland, brain, colon, heart, leukocytes, pancreas, prostate, salivary gland, skeletal muscle, small intestine, smooth muscle, and thyroid) were obtained as purified RNAs from Takara Bio.

### Experimental animals

*Prss54* knockout mice were produced at Baylor College of Medicine, and *Prss44* and *Prss46* knockout mice were produced at Osaka University, Japan. B6D2F1 (C57BL/6 × DBA2) mice were used as embryo donors and CD1 (BCM) or ICR (Osaka) mice were used as foster mothers. Mice were purchased from either Charles River (Wilmington, MA, USA) or Japan SLC (Shizuoka, Japan). All mice were housed in a temperature-controlled environment with 12 h light cycles and free access to food and water.

### Generation of *Prss54* knockout mice

To generate *Prss54* knockout mice, gRNA/Cas9 ribonucleoprotein complex was electroporated into fertilized eggs and transplanted into surrogate mothers as previously described [42]. Briefly, to harvest fertilized eggs, CARD HyperOva (0.1 mL, Cosmo bio) was injected into the abdominal cavity of B6D2F1 females (Charles River), followed by human chorionic gonadotropin (hCG) (5 units, EMD Chemicals). Forty-eight hours after CARD HyperOva, B6D2F1 males were allowed to mate naturally. Twenty hours after mating, fertilized eggs with two pronuclei were collected for electroporation. Custom crRNA targeting Exon 1 of *Prss54* was purchased from Milipore-Sigma. The sequences for all guide RNAs used for CRISPR/Cas9-mediated gene editing are listed in Supplementary Table S3. crRNA and tracrRNA (Milipore-Sigma) were diluted with nuclease-free water. The mixture was denatured at 95 °C for 5 min and allowed to anneal by cooling gradually to room temperature (1 h). Each gRNA was mixed with Cas9 protein solution (Thermo Fisher Scientific) and opti-MEM media (Thermo Fisher Scientific), and then incubated at 37 °C for 5 min to prepare the gRNA/Cas9 RNPs (final concentration: 300 ng/μL Cas9 for 250 ng/μL of each gRNA). The gRNA/Cas9 RNP solution was placed between electrodes with a 1-mm gap in the ECM 830 Electroporation System (BTX). Fertilized eggs were arranged between the electrodes and then the electroporation was performed with the following conditions: 30 V, 1-ms pulse duration, 2 pulses separated by 100-ms pulse interval. For egg transfer, electroporated embryos were transplanted into the oviduct of pseudo-pregnant ICR recipients. After 19 days, offspring were obtained by natural birth or Caesarean section. The F0 mice with sequence-predicted heterozygous mutations were used for the mating with C57/129 mice to generate homozygous mutants. The F2 or later generation was used for the phenotypic analysis.

### Generation of *Prss44* and *Prss46* knockout mice

The CRISPR/Cas9-mediated genome editing in embryonic stem cells was previously shown to be an effective and rapid method of generating targeted mutations in mice [42]. As previously described [42], 1 × 10^3–4^ EGR-G101 embryonic stem cells (ESC) were seeded on mouse embryonic fibroblasts (MEF) in a 6-well plate and transfected with sgRNA/CAS9 expressing pX459 plasmids (total 1.0 μg) using Lipofectamine LTX & PLUS technology (Life Technologies, Carlsbad, CA, USA). After 14–18 h of transfection, the cells were selected with puromycin (0.15 μg/mL) for 48 h, then grown for 3 to 4 more days, picked, and transferred onto MEF cells in 96-well plates. After 48–72 h of culture, each ESC clone was split in duplicate, for freezing, and DNA harvesting. After PCR amplifications and direct sequencing, the positive clones were thawed and expanded to analyze their karyotypes. Approximately 6–8 mutant ESCs were injected into 8-cell stage ICR embryos, and the chimeric blastocysts were transplanted into the uteri of pseudopregnant females.

### Genotype analysis of *Prss44*, *Prss46*, and *Prss54* knockout mice

Genomic DNA was isolated from mutant mice by incubating tail tips in lysis buffer (20 mM Tris–HCl [pH 8.0], 5 mM EDTA, 400 mM NaCl, 0.3% SDS, and 200 μg/mL actinase E solution) at 60 °C overnight. PCR was performed using KOD Xtreme or KOD FX neo (TOYOBO, Osaka, Japan). Primer sets used for PCR are listed in Supplementary Table S4. PCR products were purified using Wizard SV Gel and PCR Clean-Up System (Promega, Madison, WI, USA) and Sanger sequenced with an ABI 3130x/Genetic Analyzer (Thermo Fisher Scientific, Waltham, MA, USA) using the antisense primer. Oligonucleotides were purchased from Milipore-Sigma (Woodlands, TX) or GeneDesign (Osaka, Japan).

### Male fertility analysis

Upon sexual maturation, knockout and control male mice (*n* ≥ 3 per genotype) were continuously housed with two 7–8-week-old wild-type B6D2F1/J female mice for at least 8 weeks. During the fertility test, the number of pups was counted at birth. The average litter size for each mouse line was calculated by dividing the total number of pups with the number of litters.

### Testis weights and testis and epididymis histology of male mice

After the fertility test, knockout and same-aged control male mice (*n* ≥ 3 per genotype) were euthanized by cervical dislocation following by anesthesia to examine testis weights, testicular and epididymal histology, sperm morphology, and sperm motility. Testes and epididymides were fixed in Bouin fixative, embedded in paraffin, sectioned at 5 μm thickness, and stained with 1% periodic acid Schiff (PAS) stain followed by counterstaining with Mayer hematoxylin solution.

### Sperm analysis

For *Prss54* knockout and control mice (*n* = 8 per genotype), the cauda of both epididymides were isolated and transferred into Human Tubule Fluid (HTF) (Irvine Scientific, Santa Ana, CA) containing 5 mg/mL of BSA. The cauda were minced and placed in a humidified incubator for 15 min at 37 °C with 5% CO_2_. Following incubation, the sperm were diluted 1:50 in HTF, added to a prewarmed slide, and analyzed with computer-assisted sperm analysis (CASA) using Hamilton-Thorne Bioscience’s Ceros II software program. Several (*n* ≥ 6) fields of view were illuminated and captured until at least 600 cells were counted.

**Figure 1:**
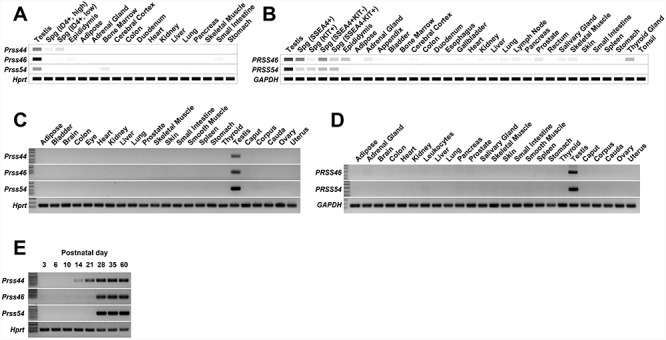
Patterns of gene expression in mouse and human tissues and organs. **(A)** Digital PCR depicting the average TPM value per tissue per gene from 77 published mouse RNAseq datasets. White = 0 TPM, Black ≥ 30 TPM. **(B)** Digital PCR depicting the average TPM value per tissue per gene from 147 published human RNAseq datasets. White = 0 TPM, Black ≥ 30 TPM. **(C)** Conventional RT-PCR of mouse tissues and organs. **(D)** Conventional RT-PCR of human tissues and organs. **(E)** Conventional RT-PCR of mouse testes isolated at the developmental time points.

**Figure 2:**
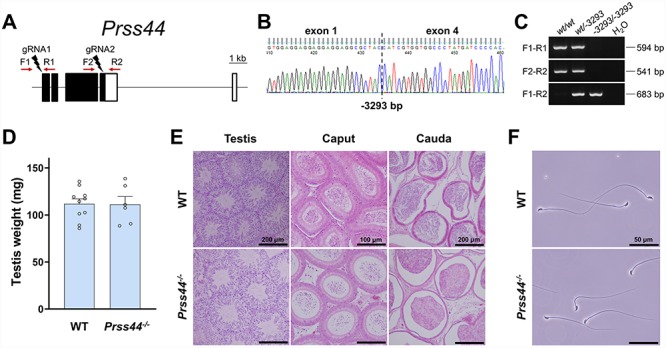
Phenotypic analysis of *Prss44* knockout male mice. **(A)** Genomic structure and knockout strategy of mouse *Prss44*. Dual sgRNAs were designed to target the first and last coding exons. F1/F2, forward primer for genotyping; R1/R2, reverse primers for genotyping. **(B)** Genotype validation of *Prss44* knockout mice by Sanger sequencing. **(C)** Genotype validation of *Prss44* knockout mice by genomic PCR. **(D)** Comparison of testis weights of *Prss44^+/+^* wildtype (WT) and *Prss44^−/−^* knockout (KO) mice. **(E)** Histological analyses of testes and epididymides in *Prss44* WT and KO mice. **(F)** Morphology of spermatozoa extracted from the cauda epididymides of *Prss44* WT and KO mice. Scale bars as indicated.

**Figure 3:**
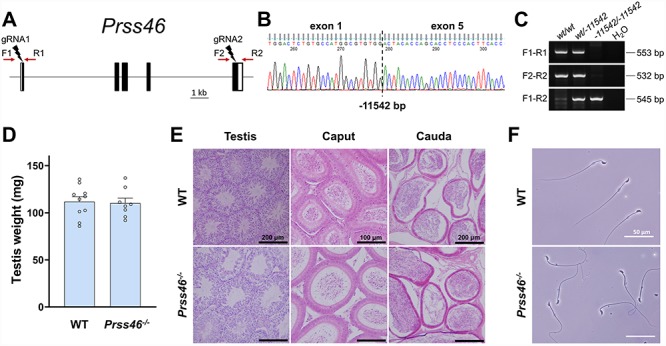
Phenotypic analysis of *Prss46* knockout male mice. **(A)** Genomic structure and knockout strategy of mouse *Prss46*. Dual sgRNAs were designed to target the first and last coding exons. F1/F2, forward primer for genotyping; R1/R2, reverse primers for genotyping. **(B)** Genotype validation of *Prss46* knockout mice by Sanger sequencing. **(C)** Genotype validation of *Prss46* knockout mice by genomic PCR. **(D)** Comparison of testis weights of *Prss46^+/+^* wildtype (WT) and *Prss46^−/−^* knockout (KO) mice. **(E)** Histological analyses of testes and epididymides in *Prss46* WT and KO mice. **(F)** Morphology of spermatozoa extracted from the cauda epididymides of *Prss46* WT and KO mice. Scale bars as indicated.

### Statistical analysis

All measurements are expressed as mean ± standard error of the mean. Statistical differences were determined using the Student *t*-test. Differences were considered statistically significant if the *P* value was less than 0.05. All statistical analyses were conducted using Microsoft Office Excel (Microsoft Corporation, Redmond, WA, USA).

## Results

### Expression of *Prss44*, *Prss46*, and *Prss54*

Two hundred and twenty-four previously published [37–41] human and mouse RNAseq datasets were processed in parallel through a custom bioinformatic pipeline designed to identify novel reproductive tract-specific and -enriched transcripts. We identified *Prss46* and *Prss54* as congruent in expression across both mouse and human datasets with expression restricted to testes and spermatogenic cells (Figure 1A and B). *Prss44* was also identified in mouse with similar testis-restricted expression (Figure 1A); however, in humans, *PRSS44* is an unprocessed pseudogene and not expressed. Conventional RT-PCR of a panel of mouse and human tissue cDNAs confirmed testis-restricted expression of *Prss46* and *Prss54* in both species and *Prss44* in mice (Figure 1C and D). To glean insight into the potential spermatogenic cell population(s) expressing each of these genes, we performed RT-PCR of mouse testes isolated at postnatal day (P) 3, a timepoint enriched for gonocytes, P6 (onset of expression of Type A spermatogonia), P10 (early spermatocytes), P14 (late spermatocytes), P21 (spermatids), and P35 and P60, which display complete spermatogenesis [43]. Expression of *Prss46* and *Prss54* is detected at similar levels at P28 and later, but not at P21 or before indicating expression during spermiogenesis and spermiation. Expression of *Prss44* begins earlier, with a gradual increase beginning at P14, suggesting a potentially different role for this protease during spermatogenesis.

### In vivo analyses of *Prss44*, *Prss46*, and *Prss54*

**Figure 4:**
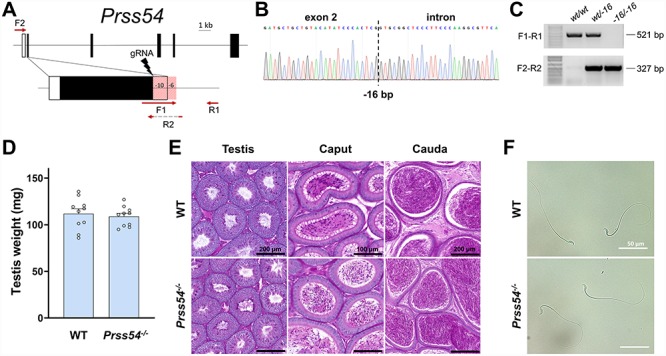
Phenotypic analysis of *Prss54* knockout male mice. **(A)** Genomic structure and knockout strategy of mouse *Prss54*. Since sgRNAs was designed to target the first coding exons. F1/F2, forward primer for genotyping; R1/R2, reverse primers for genotyping. **(B)** Genotype validation of *Prss54* knockout mice by Sanger sequencing. **(C)** Genotype validation of *Prss54* knockout mice by genomic PCR. **(D)** Comparison of testis weights of *Prss54^+/+^* wildtype (WT) and *Prss54^−/−^* knockout (KO) mice. **(E)** Histological analyses of testes and epididymides in *Prss54* WT and KO mice. **(F)** Morphology of spermatozoa extracted from the cauda epididymides of *Prss54* WT and KO mice. Scale bars as indicated.

To determine the male reproductive requirement and potential functional role of the identified serine proteases, each gene was individually ablated by a CRISPR/Cas9-mediated ES cell or zygote approach. The efficiency of generating each mutant is summarized in Supplementary Table S5. Each of the four genes contained deletions of differing sizes and genomic targets. For *Prss44* and *Prss46*, the gene loci were mostly deleted with dual sgRNAs that were designed to target the first and last coding exons (Figures 2A and 3A). For *Prss54*, the gene was disabled by introducing an indel in the first coding exon using only one sgRNA (Figure 4A). The genomic sequences flanking the deletion in each mutant is presented in Supplementary Table S6 and representative sanger sequencing results for each mutant are presented in Figures 2B, 3B, and 4B. Using the forward and reverse primer pairs presented in Figures 2A, 3A, and 4A, and listed in Supplementary Table S4, offspring carrying the *Prss44*, *Prss46*, and *Prss54* knockout alleles were identified through routine genotyping (Figures 2C, 3C, and 4C).

**Table 1:** Outcomes of the fertility tests for the four knockout mouse lines.

Gene symbol	Genotype	No. of males	No. of pups	No. of litters	Mating period	Average litter size ±SD
Wildtype	+/+	3	180	20	10 weeks	9.0 ± 2.7
*Prss44*	−3293/−3293	3	219	27	15 weeks	8.1 ± 2.7
*Prss46*	−11 542/−11 542	3	128	16	9 weeks	7.8 ± 3.4
*Prss54*	−16/−16	4	128	17	8 weeks	7.1 ± 2.3

Each of the knockout mouse lines was examined in parallel with littermate wildtype controls of equivalent age to determine the effect of gene ablation on spermatogenesis, sperm maturation, and fertility in male mice. None of the knockout strains generated in this study displayed any overtly abnormal appearance, difference in body weight or composition, or difference in behavior when compared to the controls. The testes weights of *Prss44*, *Prss46*, and *Prss54* knockout mice were not significantly different from control mice (Figures 2D, 3D, and 4D). Histological analyses of testes from *Prss44*, *Prss46*, and *Prss54* knockout mice revealed intact seminiferous epithelia with all germ cell subtypes, tubules at all stages of spermatogenesis, and no histological defects in comparison to control testis sections (Figures 2E, 3E, and 4E). Histological analyses of caput and cauda epididymides revealed spermatozoa in tubule lumens and no significant differences in comparison to controls (Figures 2E, 3E, and 4E). Cauda epididymal sperm isolated from mutant animals looked morphologically indistinguishable to controls (Figures 2F, 3F, and 4F), and CASA of *Prss54* knockouts showed no statistical differences across all measured parameters (Supplementary Table S7).

### Fertility tests for knockout male mice

To determine the male reproductive requirement of each of the genes of interest, *Prss44*, *Prss46*, and *Prss54* knockout and control adult male mice were housed continuously with two females for at least 2 months and the size and number of litters was recorded. Knockout males sired a number and size of litters during the test mating period that was not significantly different from controls (Table 1).

## Discussion

Although many testis-specific genes have been studied through functional genetics approaches, many remain to be solved. Elucidating the function of these novel genes is necessary to build a better understanding of the factors underlying spermatogenesis and sperm maturation, which has implications in understanding the etiology of male infertility and the development of male contraceptives. In this study, the testis-specific proteases, *Prss44*, *Prss46*, and *Prss54* were deleted in mice to determine their functional requirement in male fertility.

Considering testes sizes were normal in *Prss44*, *Prss46*, and *Prss54* knockout lines, and the expression of these genes does not begin until after the initiation of meiosis (Figure 1E), a requirement for these genes during gonad development is unlikely. Analyses of testis and epididymis histology, sperm morphology, and fertility revealed no significant differences in *Prss44*, *Prss46*, and *Prss54* knockout mice in comparison to controls. While future investigations may find subtle phenotypic changes in these knockout mice, we have shown that, individually, *Prss44*, *Prss46*, and *Prss54* are not a requirement for male mouse fertility. Although each of the generated mouse models exhibited normal fertility, we cannot rule out the possibility of functional redundancy. Generation of mutant mice containing simultaneous loss of two or more serine protease genes may reveal an infertility phenotype and functional requirement for this gene group. The loss of function of a gene when a knockout model is created can be compensated by paralogous genes that contribute to a single function [44]. For example, it is plausible that the presence of the paralogs *Prss21*, *Prss37*, and/or *Prss55*, in the absence of *Prss44*, *Prss46*, and *Prss54*, could generate robustness of function against the gene deletion. In addition, orthologs may also be an area of interest for further investigation.

Orthologs are produced through speciation. As evolution progresses, the dynamic nature of DNA replication can result in changes of genomic composition, resulting in pseudogenes. Pseudogenes are genes that have lost their protein-coding ability. Factors such as frameshift mutations and sequence decay within the genome may lead to malformed DNA causing a loss of elements needed for transcription or translation [45]. In our study, we encountered two genes of interest that in mice are protein coding, but in humans are pseudogenes, *PRSS44* and *PRSS46.* In humans, *PRSS44* is an unprocessed pseudogene, while *PRSS46* is a processed pseudogene. Unprocessed pseudogenes are genes that lack the ability to make transcripts, while processed pseudogenes, although make transcripts, lack the ability to make proteins [46]. While translation of these transcripts in humans does not occur, further study of these genes may reveal differences in their roles in both species. Furthermore, lack of protein-coding ability does not necessarily indicate that these genes are functionally obsolete. Pseudogenes have been shown to play roles in gene expression and gene regulation [46]. For example, pseudogene transcripts can act as competitive endogenous RNAs (ceRNAs) through competitive binding of miRNA, which results in regulation of gene expression [47]. To this end, studying the functional requirement of *Prss44* and *Prss46* in male mice is necessary to further our knowledge of evolutionarily conserved genes between species.

In summary, knockout models for *Prss44*, *Prss46*, and *Prss54* were generated through CRISPR/Cas9 to determine their male reproductive requirement. Each of the male models exhibited normal fertility and did not display any noticeable phenotypic changes. Although we did not identify the specific functions or localizations of these serine proteases, it is essential to disseminate to the scientific community that *Prss44*, *Prss46*, and *Prss54* are not required for male fertility, which can prevent costly repetition and duplication of efforts by other laboratories.

## Supplementary Material

BIOLRE-2019-0106_Supplementary_Figures_revised_noPRSS58_ioz158
