# Supplementary material for: The testis-specific serine proteases PRSS44, PRSS46, and PRSS54 are dispensable for male mouse fertility
Source: Biol Reprod. 2019 Aug 12;102(1):84–91. doi: 10.1093/biolre/ioz158 (PMC7013879; doi:10.1093/biolre/ioz158)
Supplement: BIOLRE-2019-0106_Supplementary_Figures_revised_noPRSS58_ioz158 [file biolre-2019-0106_supplementary_figures_revised_noprss58_ioz158.docx]

**Table S1.** Sequence Read Archive (SRA) values for all of the dataset used in this study.

| **Human Tissue** | **Samples (N)** | **SRA Run #** |
| --- | --- | --- |
| adipose tissue | 5 | ERR315332, ERR315343, ERR315342, ERR315378, ERR315431 |
| adrenal gland | 5 | ERR315335, ERR315392, ERR315417, ERR315385, ERR315452 |
| appendix | 5 | ERR315345, ERR315366, ERR315437, ERR315465, ERR315481 |
| bladder | 5 | ERR315334, ERR315370, ERR315355, ERR315447, ERR315421 |
| bone marrow | 5 | ERR315333, ERR315395, ERR315425, ERR315396, ERR315406 |
| colon | 5 | ERR579129, ERR315348, ERR315357, ERR315403, ERR315400 |
| cerebral cortex | 3 | ERR315432, ERR315455, ERR315477 |
| duodenum | 4 | ERR315442, ERR315445, ERR315457, ERR315461 |
| heart | 5 | ERR315328, ERR315331, ERR315384, ERR315367, ERR315356 |
| gallbladder | 5 | ERR315349, ERR315347, ERR315360, ERR315427, ERR315474 |
| esophagus | 5 | ERR315362, ERR315434, ERR315398, ERR315411, ERR315472 |
| kidney | 4 | ERR315383, ERR315443, ERR315468, ERR315494 |
| liver | 5 | ERR315327, ERR315414, ERR315394, ERR315463, ERR315451 |
| lung | 5 | ERR315326, ERR315341, ERR315346, ERR315353, ERR315439 |
| lymph node | 5 | ERR315329, ERR315373, ERR315371, ERR315426, ERR315393 |
| pancrease | 4 | ERR315429, ERR315436, ERR315466, ERR315479 |
| prostate | 5 | ERR315330, ERR315340, ERR315359, ERR315365, ERR315354 |
| rectum | 4 | ERR579140, ERR579151, ERR579147, ERR579127 |
| salivary gland | 5 | ERR315325, ERR315382, ERR315420, ERR315418, ERR315459 |
| skeletal muscle | 3 | ERR579142, ERR579143, ERR579149 |
| skin | 5 | ERR315339, ERR315372, ERR315376, ERR315401, ERR315460 |
| small intestine | 5 | ERR315344, ERR315381, ERR315364, ERR315409, ERR315419 |
| spleen | 5 | ERR315338, ERR315416, ERR315448, ERR315405, ERR315473 |
| stomach | 4 | ERR315369, ERR315379, ERR315485, ERR315467 |
| thyroid gland | 5 | ERR315337, ERR315358, ERR315363, ERR315428, ERR315397 |
| tonsil | 2 | ERR579133, ERR579139 |
| testis | 5 | ERR315351, ERR315352, ERR315350, ERR315391, ERR315446 |
| epididymis (Cauda) | 2 | SRR2392510, SRR2392509 |
| epididymis (Corpus) | 2 | SRR2392508, SRR2392507 |
| epididymis (Caput) | 2 | SRR2392506, SRR2392505 |

**Table S1.** (cont’d)

| **Human Cells** | **Samples (N)** | **SRA Run #** |
| --- | --- | --- |
| spermatogonial stem cells (SSEA4-/KIT+) | 6 | [SRR5099528, SRR5099527, SRR5099518, SRR5099517, SRR5099516, SRR5099515](https://www.ncbi.nlm.nih.gov/Traces/sra/?run=SRR5099527) |
| spermatogonial stem cells (SSEA4+/KIT-) | 4 | SRR5099526, SRR5099525, SRR5099520, SRR5099519 |
| spermatogonial stem cells (SSEA4+) | 6 | SRR5099524, SRR5099523, SRR5099514, SRR5099513, SRR5099512, SRR5099511 |
| spermatogonial stem cells (KIT+) | 2 | SRR5099522, SRR5099521 |
|  |  |  |
| **Mouse Tissue** | **Samples (N)** | **SRA Run #** |
| adipose tissue | 4 | SRR5048011, SRR5048013, SRR5048012, SRR5048014 |
| adrenal gland | 5 | SRR5047957, SRR5047958, SRR5047959, SRR5047960, SRR5047961 |
| bone marrow | 2 | SRR5171014, SRR5171015 |
| colon | 5 | SRR5047913, SRR5047914, SRR5047915, SRR5047916, SRR5047917 |
| cerebral cortex | 4 | SRR3191882, SRR3191883, SRR3191884, SRR3191885 |
| duodenum | 5 | SRR5047963, SRR5047964, SRR5047965, SRR5047966, SRR5047967 |
| heart | 5 | SRR5047921, SRR5047922, SRR5047923, SRR5047924, SRR5171028 |
| kidney | 5 | SRR5047925, SRR5047926, SRR5047927, SRR5047928, SRR5047929 |
| liver | 5 | SRR5047931, SRR5047932, SRR5047933, SRR5047934, SRR5047935 |
| lung | 5 | SRR5047937, SRR5047938, SRR5047939, SRR5047940, SRR5171030 |
| pancrease | 2 | SRR5171086, SRR5171087 |
| skeletal muscle | 5 | SRR3191889, SRR3191890, SRR3191891, SRR3191892, SRR3191893 |
| small intestine | 5 | SRR5048010, SRR5171046, SRR5171047, SRR5171080, SRR5171081 |
| stomach | 5 | SRR5047995, SRR5047996, SRR5047997, SRR5047998, SRR5047999 |
| testis | 9 | SRR5047953, SRR5047954, SRR5047955, SRR5047956, SRR5171084, SRR5171085, GSM2464293, GSM2464296 |
|  |  |  |
| **Mouse Cells** | **Samples (N)** | **SRA Run #** |
| high-ID4_spermatogonia | 3 | GSM2464299, GSM2464301, GSM2464303 |
| low-ID4_spermatogonia | 3 | GSM2464300, GSM2464302, GSM2464304 |

**Table S2.** Primers used for RT-PCR.

| **Gene** | **Species** | **Forward Primer** | **Reverse Primer** |
| --- | --- | --- | --- |
| *Prss44* | Mouse | GTATGGTTCCAGGGACGCC | TGGCCACAAGCTGAAGTGGG |
| *Prss46* | Mouse | TCAGTGGATCCTCACGGTCT | ACCAGATCCAGGGACCTTCC |
| *Prss54* | Mouse | TACCTTCTCTCCTTGGGCCATC | GTGGGTGTACTGCTTGTCCT |
| *Hprt* | Mouse | GTTGGGCTTACCTCACTGCT | TCATCGCTAATCACGACGCT |
| *PRSS46* | Human | ACATCTCCGCTTTCCTCTGC | ACTCCCACCATCACGGAGTA |
| *PRSS54* | Human | TGCAGCATTTTATGGCTACAGA | ATGGGCAGCTGTCTTATCCC |
| *GAPDH* | Human | AATCCCATCACCATCTTCCAG | ATGACCCTTTTGGCTCCC |

**Table S3.** sgRNAs used for generating knockout mice. *Prss44*, *Prss46*, and *Prss58* were done through ES approach meanwhile *Prss54* was done through zygote approach.

|  | **Strand** | **sgRNA target sequence** | **PAM** (NGG) |
| --- | --- | --- | --- |
| *Prss44* | **5’** | GGAGGAGGAGGCGCTACAAC | AGG |
|  | **3’** | ATAGGGCCACCACGATGGCC | AGG |
| *Prss46* | **5’** | TGCCATGGCGTGTGGATCAG | TGG |
|  | **3’** | GAGGTGCTGGTGTAGACGCT | TGG |
| *Prss54* | **5’** | CTCGTCATCTGCCAGTGAGT | CCA |

**Table S4.** Primers and PCR conditions used for genotype validation of CRISPR/Cas9-derived mutant mice.

|  |  | **Forward primer** | **Reverse primer** | **Annealing conditions** | **Elongation conditions** | **Band size** (bp) |
| --- | --- | --- | --- | --- | --- | --- |
| *Prss44* | **WT** | GCACCCTGTCGCAGTGATTA | ACCCGAGTCATCTAGGCCAT | 65 C, 45s | 72 C, 30s | 594 |
|  | **WT** | GAGCCTTGGGGTGGAATCTA | GTGGGCCACAATCCTTCACT | 65 C, 45s | 72 C, 30s | 542 |
|  | **KO** | GCACCCTGTCGCAGTGATTA | GTGGGCCACAATCCTTCACT | 65 C, 45s | 72 C, 30s | 683 |
| *Prss46* | **WT** | TAGGCATGAAGGCCTTTGCT | CTGGTGCCACTCTGTATGGG | 65 C, 45s | 72 C, 30s | 553 |
|  | **WT** | AGGATGGCTCAGTGGTCAGA | CTCCGGTGTCCACGTCTTCT | 65 C, 45s | 72 C, 30s | 532 |
|  | **KO** | TAGGCATGAAGGCCTTTGCT | CTCCGGTGTCCACGTCTTCT | 65 C, 45s | 72 C, 30s | 545 |
| *Prss54* | **WT** | TATCCCACTCGTCATCTGCCAGTGAGT | AGCTGTCCCTTGTTTGTCTGC | 60°C, 30s | 72°C, 30s | 521 |
|  | **KO** | CCTTCTCTCCTTGGGCCATC | AAGGGAGCCGCACCGAGT | 60°C, 30s | 72°C, 30s | 327 |

**Table S5.** Efficiency of embryo transplantation and genome editing.

|  | **Picked-up clones** | **Clones with KO allele** | **Injected ESC clones** | **Injected embryos** | **Recipient** | **Pup** | **Male chimera** |
| --- | --- | --- | --- | --- | --- | --- | --- |
| *Prss44* | 24 | 14 | 2 | 60 | 4 | 8 | 6 |
| *Prss46* | 10 | 5 | 2 | 60 | 4 | 26 | 10 |

|  | **Concentration** | |  |  |  |  |  |  |  |  |
| --- | --- | --- | --- | --- | --- | --- | --- | --- | --- | --- |
|  | **gRNA** | **Cas9** | **electroporated eggs** | | **survived** | **transfered** | **mother** | **pregnant** | **pups born** | **mutant pups** |
| *Prss54* | 300 ng/ul | 250 ng/ul | Zona (+) | 149 | 126 | ND | 3 | 2 | 7 | 4 |

**Table S6.** Detailed genotype of CRISPR/Cas9-derived mutant mice determined by Sanger sequencing.

| **Gene** | **Upstream sequence** (25 bp) | **Mutation** | **Downstream sequence** (25 bp) |
| --- | --- | --- | --- |
| *Prss44* | GTGGAGGAGGAGGAGGAGGCGCTAC | -3293 | CATCGTGGTGGCCCTATGATCCCCA |
| *Prss46* | TGGACTCTGTGCCATGGCGTGTGGA | -11542 | CTACACCAGCACCTCCCACTTCACC |
| *Prss54* | GATGCTGCTGTACATATCCCACTCG | -16 | GTGCGGCTCCCTTCCCAAGGCGTTC |

**Table S7.** Computer-assisted sperm analysis (CASA) results of control and *Prss54^-/-^* knockout mice. Smoothed path velocity (VAP); Curvilinear velocity (VCL); Straight line velocity (VSL); Amplitude of lateral head displacement (ALH); Beat cross frequency (BCF); Linearity (LIN) (VSL/VCL); Straightness (STR) (VSL/VAP) ; Wobble (WOB)

|  | **Control (n=8 mice)** | **Prss54-/- (n=8 mice)** | **P-value** |
| --- | --- | --- | --- |
| **Total Cells (x10^6^)** | 31.49±3.05 | 30.11±4.12 | 0.79 |
| **% Motility** | 29.86±3.07 | 24.09±4.34 | 0.3 |
| **% Progressive Motility** | 24±3.44 | 20.08±3.69 | 0.45 |
| **% Static** | 70.14±3.07 | 75.91±4.34 | 0.3 |
| **% Hyperactivated** | 1.38±0.31 | 1.58±0.34 | 0.67 |
| **VAP (µm/sec)** | 141.84±7.48 | 131.95±8.9 | 0.41 |
| **VCL (µm/sec)** | 242.25±12.12 | 218.82±11.38 | 0.18 |
| **VSL (µm/sec)** | 122.62±7.15 | 116.32±8.22 | 0.57 |
| **ALH (µm)** | 11.46±0.7 | 10.28±0.68 | 0.24 |
| **BCF (beats/sec)** | 29.73±1.19 | 27.58±0.72 | 0.14 |
| **LIN (%)** | 51.54±2.07 | 54.67±2.1 | 0.31 |
| **STR (%)** | 85.79±1.56 | 87.51±0.93 | 0.36 |
| **WOB (%)** | 59.36±1.49 | 61.6±1.87 | 0.36 |
